# Concrete Performance Attenuation of Mix Nano-SiO_2_ and Nano-CaCO_3_ under High Temperature: A Comprehensive Review

**DOI:** 10.3390/ma15207073

**Published:** 2022-10-11

**Authors:** Deprizon Syamsunur, Li Wei, Zubair Ahmed Memon, Salihah Surol, Nur Izzi Md Yusoff

**Affiliations:** 1 Department of Civil Engineering, Faculty of Engineering, Technology and Built Environment, UCSI University, Kuala Lumpur 56000, Malaysia; 2 Postgraduate Studies, Universitas Bina Darma Palembang, Kota Palembang 30111, South Sumatera, Indonesia; 3 College of Engineering, Prince Sultan University, Riyadh 11586, Saudi Arabia; 4 Department of Civil Engineering, Faculty of Engineering and Built Environment, Universiti Kebangsaan Malaysia, Bangi 43600, Malaysia

**Keywords:** composite nanomaterials, high-temperature environment, concrete structures, performance decay

## Abstract

Fire and extreme heat environmental changes can have an impact on concrete performance, and as climate change increases, new concrete structures are being developed. Nano-silica and nano-calcium carbonate have shown excellent performances in modifying concrete due to their large specific surface areas. This review describes the changes in concrete modified with nano-silica (NS) and nano-calcium carbonate (NC), which accelerate the hydration reaction with the cementitious materials to produce more C-S-H, resulting in a denser microstructure and improved mechanical properties and durability of the concrete. The mechanical property decay and visualization of deformation of mixed NS and NC concrete were tested by exposure to high temperatures to investigate the practical application of mixed composite nanomaterials (NC+NS) to concrete. The nano-modified concrete had better overall properties and was heated at 200 °C, 400 °C, 600 °C and 800 °C to relatively improve the mechanical properties of the nano concrete structures. The review concluded that high temperatures of 800 °C to 1000 °C severely damaged the structure of the concrete, reducing the mechanical properties by around 60%, and the dense nano concrete structures were more susceptible to cracking and damage. The high temperature resistance of NS and NC-modified nano concrete was relatively higher than that of normal concrete, with NC concrete being more resistant to damage at high temperatures than the NS samples.

## 1. Introduction

**Figure 1:**
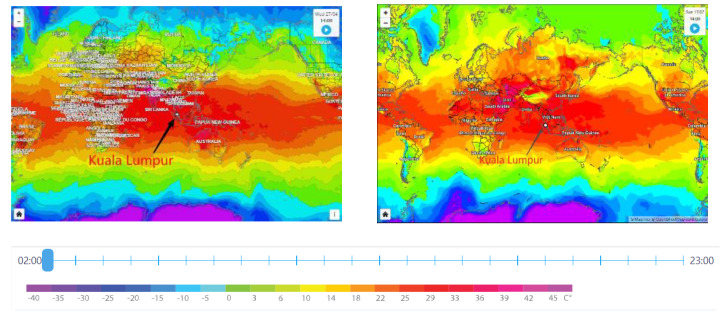
Maps of global temperature zone, (source: https://www.foreca.com/, accessed on 15 August 2022).

Cement concrete has long been a structural material in civil engineering and continues to give rise to concepts such as Ultra-High-Performance Concrete (UHPC) [1,2], Recycled Aggregate Concrete [3] and Fibre-Reinforced Concrete [4,5], proposing 3D Printed Concrete Technology [6] for new types of construction and driving high-quality development in the engineering industry [7,8,9,10,11]. Although concrete is a durable material, the internal structure and materials are subject to various environmental extremes over time [12], such as high-temperature environments caused by warming [13], especially dense nano concrete, and these damages have important implications for today’s sustainable development [14,15,16,17,18]. Peninsular Malaysia and East Malaysia are closer to the equator in latitude and are exposed to high-temperature environments year-round [19]. In the past decade, the ambient temperature has shown an increasing trend [20]. The long-term average temperature in Malaysia has increased by 0.5–1.5 °C over the past three decades and 0.5–1.0 °C in the past decade [21]. As a result of climate change, significant temperature increases are expected in Peninsular Malaysia and East Malaysia over the next 100 years [22]. Maps of the global temperature zone are shown in Figure 1, dated 27 April 2022 and 17 July 2022.

NS and NC are common nanomaterials in the market and are used in a wide variety of applications [23]. NS has a multi-stage hydration reaction within the cementitious material and acts mainly in the pre-concrete phase [24,25]. In contrast, NC materials are inert, with smaller particles filling the tiny voids within the concrete and having a long-term effect on concrete properties [26,27,28]. The market pricing of NS is higher than NC due to differences in preparation and source [29]. To some extent, nanomaterial concrete applications raise the economic cost of the project [30]. By exploring the performance benefits of nano concrete and justifying the decay in extreme temperature environments, a balance line can be identified to promote the durable and sustainable development of the engineering industry [23,31].

The addition of NS improves the performance of concrete and can significantly improve the defects that exist in the concrete itself [32]. NS accelerates the hydration within the concrete cementitious material, generating additional hydration products, and the dense microstructure increases the strength of the frictional bond between the fibres and the matrix, improving the mechanical properties of the concrete matrix [27,33]. The transition zone between the concrete aggregate and the cement matrix is usually the weakest. NS micronises the concrete interface transition zone and reduces the width of wear cracks at the interface transition zone [34]. By reducing the water–cement ratio of concrete, a portion of the hydration products between the aggregate and the cement matrix will overlap each other. The denseness of the interfacial transition zone is more pronounced with the addition of nano-silica [35]. NC can be used as a filler to make the microstructure of concrete denser. The study of NC and fibrous concrete in combination with 3D printer technology found that the addition of fibres and NC enhanced shape retention and resulted in better buildability [36]. NC reduced the fluidity of the concrete, but the samples with NC added improved the mechanical strength [37].

The effect of nanoparticles on the hydration of cement depends not only on the type of material but also on the temperature [38]. A certain amount of nanomaterials can effectively improve the mechanical properties of concrete, and when nano concrete is exposed to high temperatures, its internal structure and mechanisms are relatively altered. Under the temperature conditions studied of 40 °C to 70 °C, nano-SiO_2_ and nano-C-S-H shortened the induction period and the arrival time of the peak heat release rate of hydration, and nano-C-S-H also increased the peak heat release rate. The addition of nanoparticles and high temperatures led to the early production of hydrate layers [39]. Emerging concepts propose inorganic insulation nanomaterials to reduce energy use and consumption and to plan and design green buildings [40]. The science and engineering of nanotechnology have improved the high-temperature performance of concrete. The addition of nano-SiO_2_ can improve the role of the thermal insulation capacity of concrete, due to the dense internal structure, increasing the specific heat capacity and reducing the thermal diffusivity [41].

Concrete in high-temperature areas is prone to structural damage and these damages seriously affect the service life of buildings. There is a need to conduct research and develop new nanomaterial-modified concrete with good mechanical properties and durability at high temperatures. An overview of many articles found that nanomaterial concrete technology continues to be researched and developed; NC and NS-modified concrete have a good evaluation in terms of economy and performance, because the constant deterioration of extreme high-temperature environments on concrete is a long-term impact, while whether nano concrete has a lot of resistance is based on the effects of fire and internal ambient temperature. By reviewing the latest prospective research on nano concrete, it is hoped that the argumentation will provide directional guidance for future research, give a characterisation of nano concrete, make concrete structures more sustainable and promote the development of concrete technology and industrial revolution. In the future, more applications of nano concrete will be seen.

## 2. Research Methodology

**Figure 2:**
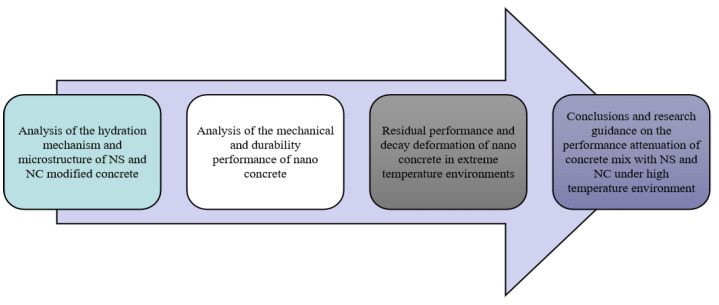
Flow chart of research methodology.

An overview of the microstructural changes, mechanical properties and durability of concrete mixed with NS and NC materials, as well as the mechanical decay and deformation in high-temperature environments is provided. Microscopic analysis techniques such as Scanning Electron Microscopy (SEM) and Differential Thermal Analyses (DTA) are used to reveal the internal morphology of the concrete. The paper reviews the changes in the mechanical properties of nano concrete in terms of compressive strength, flexural strength and durability properties such as water permeability, depth of carbonation and self-shrinkage. The final section further demonstrates the residual macro-mechanical properties and visualisation of the decaying deformation in the appearance of nano concrete after a high temperature, visualising the colour change and crack expansion of nano concrete after heating by means of content inference and picture synthesis. This review aims to investigate the effects and patterns of composite nanoparticles on the overall properties of concrete and to promote experimental research on nanocomposites (NC+NS). The following Figure 2 shows the flow chart of the research methodology for this literature review.

## 3. Nano-Modified Concrete

**Figure 3:**
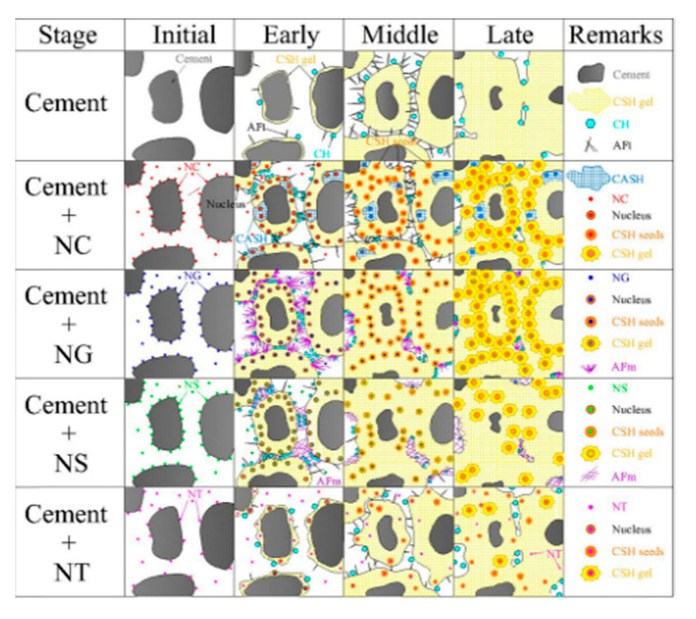
Hydration model for different nanomaterials [43].

A certain amount of nanomaterials are mixed into concrete instead of cement to accelerate the hydration and heat of the hydration reaction of cement, which helps to fill the pores in concrete. This reduces the increase in the volume and denseness of the tiny pores in concrete [42]. NS can react with the cement clinker and hydration products of cementitious materials. HSiO_2_^−4^ reacts with Ca^2+^ to produce C-S-H seeds, which undergo secondary hydration with Ca(OH)_2_ to form C-S-H gels. NC is a modifier that increases the order of CH arrangement, filling tiny voids to produce a denser cement matrix. Figure 3 shows the model of hydration of cement matrix mixed with different nanomaterials at different stages [43]. NC and fly ash blended with concrete, the seeding effect, filling effect and volcanic ash effect of the composite material contribute greatly to the mechanical properties and durability of the concrete [44].

### Microscopic Analysis

A microscopic SEM examination of the composite concrete specimens after curing indicated the internal microstructure and the degree of hydration reaction [45,46]. From the microstructure analysis, when micron calcium carbonate (MS) and NS have been added alone, a number of smaller sized crystals were formed, and the microstructure was denser. The porosity was effectively reduced with the enhanced pore size distribution when MS and NS were added into the concrete at the same time. When NS and MS were added to the concrete at the same time, a large number of smaller crystals fused with larger crystals, the texture of the mix was more closed and dense and MS and NS showed a better complementary effect on the solidification of the microstructure of the hardened cement mix, which is beneficial for durability [47]. The addition of NS and carbon nanotubes in the cement matrix provided good corrosion and water penetration resistance and showed strong resistance to carbonation in liquids with strong alkalis. The dense microstructure enhanced the performance of the durability properties [48].

**Figure 4:**
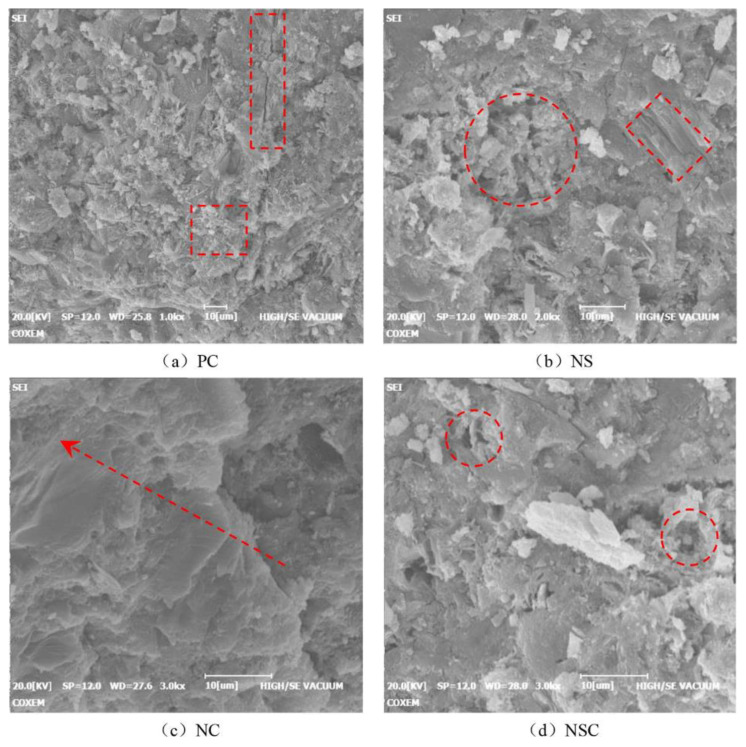
Micrographs of different additions of NC and NS [50].

Z. hang Wang et al. [49] concluded that NC can improve the denseness of concrete, optimise the pore size distribution of concrete and improve weak areas in concrete; that NS alone can cause significant weak areas within the concrete and degrade the pore structure; and that compounding NS and NC can have a synergistic effect on nanomaterials. This is different from the conclusions reached by other researchers: comparing the micrographs in Figure 4, the microstructure of the added NC is denser than that of normal cement concrete, and the SEM shows a disorganised microstructure with NS alone (see Figure 4b NS). The authors described that NS destroys the pore structure of the concrete and affects the static and dynamic mechanical properties of NS. Comparing Figure 4c with Figure 4b, the NS admixture levels of 1.5% and 2.0%, respectively, do not differ significantly. During the experiments, the authors used ultrasonic dispersion for 15 min, and the activity and internal structure of NS may be destroyed, reducing or changing the hydration reaction with the cementitious material [50,51,52]. In addition, the correlation with the brand of nanomaterials, the activity and the application of water-reducing agents, etc., should further increase or decrease the NS admixture, as concluded after extensive experimental comparative studies [53,54,55].

**Figure 5:**
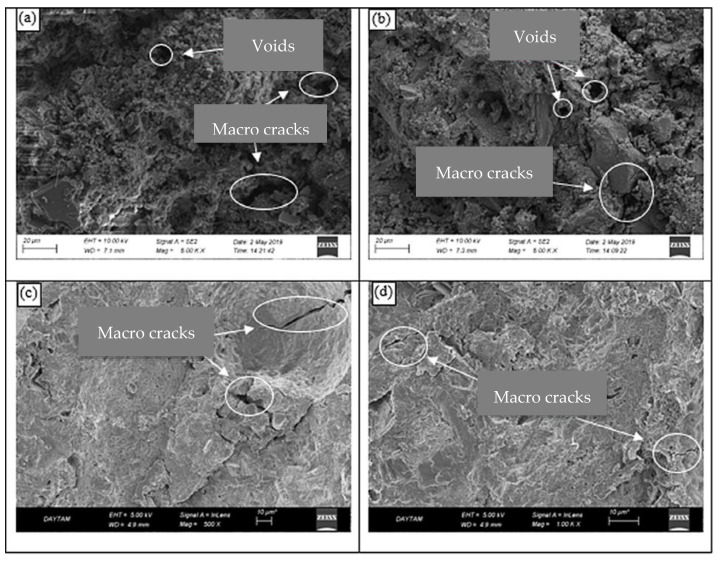
SEM micrographs of control and nano concrete samples after heating at 500 °C [31]. (**a**) Control sample, (**b**) 0.5% (NS + NA), (**c**) 1% (NS + NA), (**d**) 1.5% (NS + NA).

Guler, Türkmenoğlu et al. [31] analysed the SEM microstructure at high temperatures. The NS-doped and nano-Al2O3(NA) were heated to 300 °C, 500 °C and 800 °C. After heating at 300 °C, the internal microstructure deteriorated but still retained the compressive strength; at 800 °C the internal structure was largely destroyed, and the greatest loss of strength was observed. Many microscopic cracks and increased voids appeared inside the concrete after heating at 500 °C, as shown in Figure 5. The addition of nanomaterials at 0.5%, 1.0% and 1.5% all significantly improved the microstructure of the concrete matrix and enhanced cement paste bonding and reaction, with equivalent strength at different temperatures being higher than that of the plain samples without nanomaterials.

**Figure 6:**
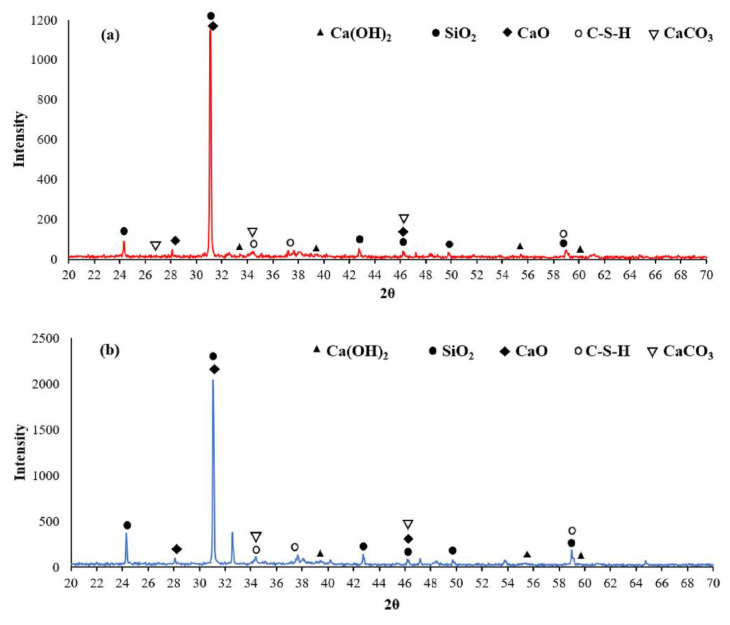
XRD spectra of (**a**) control sample and (**b**) NS sample concrete [60].

**Figure 7:**
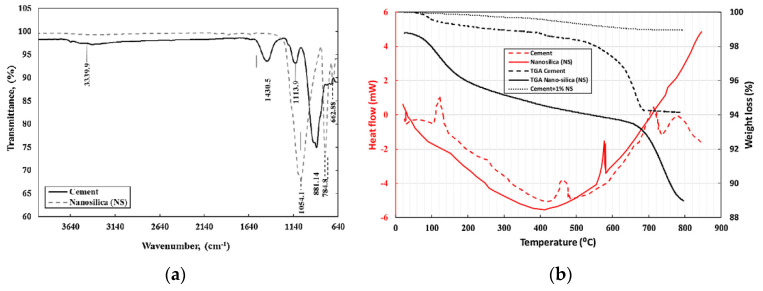
(**a**) Infrared spectrum analysis. (**b**) Thermogravimetric analysis and heat flow analysis of cement and NS [61].

**Figure 8:**
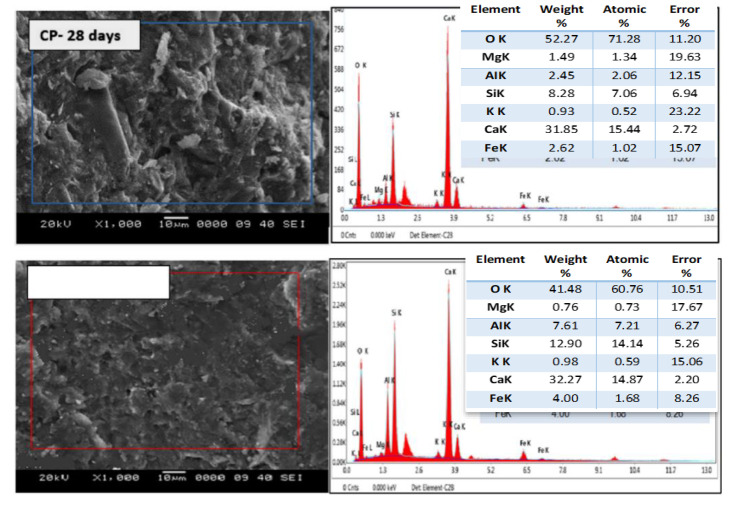
SEM-EDX analysis of control sample and 3% NS content [62].

The incorporation of NS particles at optimum dosing levels improved the structural and mechanical properties of concrete. The addition of NS increased the hydration of the cementitious material and reduced and depleted the CH content; the extent of the change can be seen in the X-ray Diffraction (XRD) patterns in Figure 6 [25,56,57,58,59]. The intensity of the CH crystal-related peaks in the nano concrete samples was reduced or disappeared compared to the conventional concrete samples. It can be concluded that CaO and CaCO3 were weaker in the samples with the NS addition, due to the higher volcanic ash activity, resulting in the elimination of the unhydrated phase CaO etc., from the cement [60]. The analysis of the infrared spectra in Figure 7a shows that NS possessed excellent properties, and that appropriate NS had a positive effect on the hydration properties of the cement paste. Figure 7b’s thermal gravimetric and heat flow analysis of cement and NS shows that below 500 °C, the heat flow of NS was higher than that of normal cement; at heating temperatures between 500 and 800 °C, the heat flow of NS was lower than the normal cement. The TGA results show that 1% silica nanoparticles additive led to low cement weight loss up to 800 °C due to the interaction of NS with cement particles in the hydrated composite and mixing the results for 1% cement + NS showed smoother results, as shown in Figure 7 [61]. The SEM-EDX of hydrated paste samples at 7 and 28 day curing ages was analysed by Snehal et al. [62]. The Ca and Si atomic content of the samples varied according to the elemental composition obtained from the EDX analysis. The SEM-EDX images of the control sample and the binary composite 3% NS are given in Figure 8. A comparison of the SEM images shows that the 3% NS replacement cement was denser. The authors used the increased Ca(OH)_3_ depletion in the 3% NS and cement binary composite samples.

## 4. The Effect of Nano-SiO_2_and Nano-CaCO_3_on the Properties of Concrete

### 4.1. Rheological Properties

The rheological properties of concrete have been evaluated accordingly by many scholars in the configuration of concrete. As NS and NC are in the nanoscale (0–100 nm) range, very small nanoparticles fill the pore structure of concrete. Nano concrete exhibits greater water absorption and a larger specific surface area, which reduce compatibility and flowability [63]. NS and NC mixed with cementitious materials produce new hydration products that fill the tiny internal voids, thus affecting the workability of the concrete. Yassoub Ahmed et al. [64] proposed that increasing the NS admixture reduced the slump results due to the extremely small particle size of NS, significantly affecting the workability of nano concrete, with higher admixture levels causing high internal agglomeration. This author utilised the use of dry mix and wet mix methods’ conclusion consistently. Nooruddin et al. [65] found that the initial and final setting times of cement mortars decreased with increasing and decreasing NS; the author used a 0.5% water–cement ratio without any type of water-reducing agent. Experiments were carried out by Mugilvani et al. [66] using a water–cement ratio of 0.45, with NS replacing cement up to 20%, 30% and 50% of the mix. To control the flow of the concrete, 20 mL of ultra-high performance water reducing agent was added during the experiments, as did the authors Danielraj et al. [67], but in the experiments, 0% to 2% NS and 10% micron-silica were used. Increasing the particle size of NS particles will inevitably change the rheological properties of nano concrete. R. Liu et al. [34] investigated the effect of changing the interfacial transition zone for different water–cement ratios on the durability properties of concrete. By reducing the experimental water–cement ratio from 0.5 to 0.35, the interfacial transition zone of the modified cementitious material of NS overlapped the reaction, which is the main reason for the reduced fluidity of the concrete. The NS modification consumed more water and also increased the water retention and cohesion to a certain extent, making the concrete more viscous in appearance. R. Liu et al. [68], comparing the durability performance of 0.4 water–cement ratio with 0.3 water–cement, found the filling and water absorption of NC caused the state of concrete to be altered. Atis et al. [69] found the reduction in the fluidity of NC concrete was due to the increase in alkali activator (NaOH) and due to the high specific surface area that NC possesses, reducing the concrete’s compatibility. There is some similarity between the filling of NS and NC particles on the alteration of rheological properties, by reducing the workability, but improving the mechanical and durability properties of the concrete with the incorporation of nanomaterials. There is a functional relationship between these properties, and according to scholarly research, it is noted that increasing the admixture of concrete increases its mechanical properties and resistance to chloride ion corrosion [70,71,72].

### 4.2. Mechanical Properties

The dynamic mechanical properties of concrete were tested by the compressive strength test, flexural strength test and splitting strength test, etc. The test process of casting cubic, rectangular and cylindrical samples can visualize the trend of a strength change of different kinds of nanomaterials in different shapes of concrete after different temperatures [73]. The incorporation of certain amounts of NC and NS materials significantly improved the mechanical properties of concrete [74,75].

**Figure 9:**
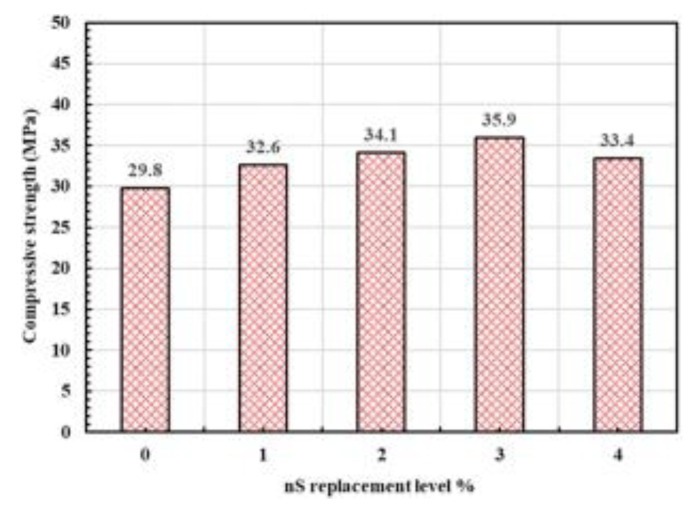
Compressive strength of different NS content at 28 days [76].

As shown in Figure 9 [76], the mechanical properties changed at 28 days for different NS admixtures, with a gradual increase in compressive strength for 1%, 2% and 3%. The compressive strength of the NS material replacing 4% cement started to decrease as the nano-doping increased. Yassoub Ahmed et al. [64] concluded that NS mixed at 1.5% had the best performance, increasing the compressive strength, flexural strength and modulus of elasticity by 28%, 57% and 62%, respectively, over the normal concrete samples. Nooruddin et al. [65] reported that mixing a small amount of NS reduced the initial and final setting time of cement mortar, and that concrete mixed with 3% NS had higher compressive and splitting tensile strengths, with increases of 22.09% and 32.19%, respectively, over the control samples. NS content greater than 3% resulted in a gradual reduction in the mechanical new properties of concrete. AlKhatib, Maslehuddin et al. [77] used cement kiln dust and electric arc furnace dust within the two industrial waste-mixed NS to develop high-performance concrete. Using 10%, 15% and 20% cement kiln dust and 10% electric arc furnace dust to replace cement, respectively, the results showed that the compressive and flexural strengths of concrete decreased, while the flexural and compressive strengths of concrete mixed with NS increased.

T. Wang et al. [78] concluded that the 1-day combined strength of UHPC increased significantly with increasing amounts of Li_2_CO_3_ and NC, and that NC was effective in mitigating the loss of the 28-day combined strength of UHPC. After modification, the UHPC combined strength and flexural strength increased by about 68% and 38%, respectively, over the control sample. The optimum dosages of 3–4% NC were obtained, and 2% and 3% of NS and NC, respectively, influenced the heat of hydration and compressive strength of the cementitious material. The addition of 2% NS had the most significant effect on the mechanical properties of the concrete [79]. The mechanical properties of concrete such as compressive strength, flexural strength and splitting strength were improved by mixing NC alone or by combining NS and NC. Mixing NS alone reduces the compressive strength, flexural strength and splitting tensile strength of concrete and can increase the modulus of elasticity of concrete [49]. The combination of NS and NC can improve the static and dynamic mechanical properties of concrete, and the effect is reduced compared to NC alone, confirming that the activity of NS is lower at this point.

According to many studies, there was no significant difference in the amount of NS and NC replacement cement, with an overall range of 0% to 3%. The findings in Table 1 below found that the use of nanomaterials mixed with different media is beneficial to the mechanical properties of concrete, with the addition of nanomaterials promoting the internal hydration of the concrete matrix, making the concrete denser and having a significant effect on the mechanical properties. The review found that the addition of nanomaterials needs to be strictly controlled in terms of optimum admixture, as excessive nanoparticle agglomeration occurs, resulting in a gradual decrease in mechanical properties, as reflected by compressive, splitting and flexural strengths. Excessive nano-doping has a detrimental effect on the structure of the concrete. Some authors suggested that the hydration effect of NS is concentrated at 7D and 28D, and that the later effect is not significant, mainly due to the volcanic ash effect and hydration reaction of the material [80,81]. On the contrary, some authors suggested that the role of NC is to enhance the mechanical properties after filling the microstructure, and its hydration effect is not strong [82], and the experimental effect of mixing NC and NS is more neutral. The authors of Z. hang Wang et al. [49] showed that mixing NC and NSC significantly improved the dynamic and static mechanical properties of nano concrete while mixing NS alone weakened them. A mixture of both was also used for both economic and performance reasons.

### 4.3. Durability

**Figure 10:**
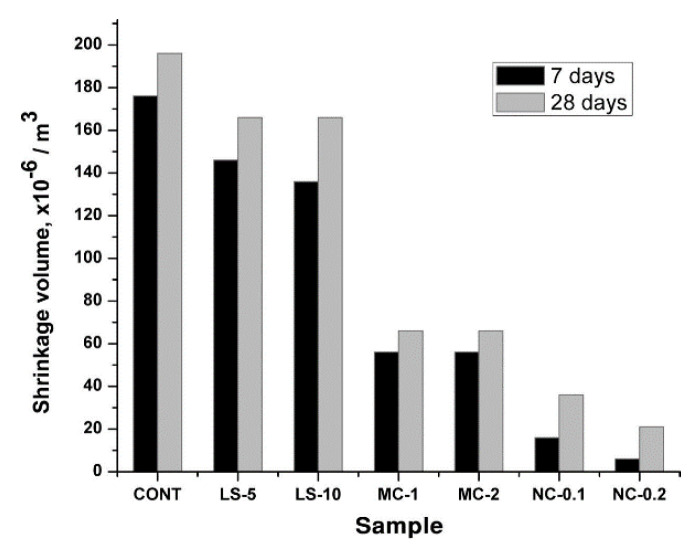
Changes in shrinkage properties of concrete after incorporation of NC [98].

NS and NC contribute significantly to the improvement of the durability properties of concrete. The addition of 1%, 2%, 3% and 4% of the cement admixture of both NS and NC can reduce the water absorption of concrete. NC makes the cement matrix microstructure denser and produces more hydration products to improve the compressive strength and durability of concrete [91,92,93]. NS has better water absorption than NC, and a more neutral mixture of composite NS and NC is the most effective [94]. This reduction is due to the presence of nanoparticle size in the pores, resulting in reduced porosity and permeability, and the optimum admixture of NS and NC mixed with blended concrete at 3% each can minimise water absorption [95]. Singh et al. [96] used concrete mixes blended with different amounts of fly ash, 3% NS particles and 6% silica fume to investigate the mechanical properties and durability of the new concrete. Compared to conventional concrete, the mixes mixed with NS showed a significant reduction in a carbonation depth of 73% after 180 days of exposure, a reduction of 39% after 180 days of erosion containing NS in sulphate and a combined specimen of fly ash and NS showed a 30% reduction. Mixing NS in concrete improved the durability and service life of the concrete. A. Zhang et al. [97] had some research results on the durability performance of NS-modified concrete. The drying process and water absorption process perspectives were investigated to test the chloride ion permeability and resistivity of concrete. Single-doped NS and compound-doped NS+nano-Al_2_O_3_ can reduce the weight loss during drying and the capillary permeability coefficient during water absorption. By increasing the nanomaterial content, the amount of charge passing through the sample is increased and the resistivity is subsequently reduced. Nanomaterial contents greater than 1% tend to agglomerate during hydration with the cementitious material, making the concrete less durable. Smaller admixtures allow for more uniform dispersion of nanoparticles and 0.5% nano concrete has better durability. In addition, the addition of NC also reduces the shrinkage of the concrete to a large extent. Figure 10 compares the change in shrinkage values between micron calcium carbonate and NC at 7 days and 28 days.

Based on the durability studies carried out by many scholars, the water absorption, hazardous material permeability and freeze-thaw resistance of nano concrete are demonstrated, and Table 2 below shows the findings for NS and NC-modified concrete. The volcanic ash effect and microfilling effect of a certain amount of NC and NS replacement cement incorporated into the concrete results in a more dense concrete, reduced water absorption and hazardous material permeability, and improves freeze-thaw resistance. Authors Nejad et al. [82] used fly ash composite-modified concrete at only 1% admixture, where nanomaterials and fly ash were used in combination, while authors H. Liu et al. [87] used NC alone to increase the testing of hydrochloric acid resistance experiments to increase the admixture of NC modification to 3% in order to obtain a better durability performance response. It is advisable to use between 2.0% and 3.0% nano-doping for bulk concrete. The review found that NS has a better ability to modify the durability properties of concrete than NC, which refines the crystalline form and enhances the structure of the interface. The modification of NS depends on the level of activity, and hydration is prominent. In addition, there is a significant difference with the water–cement ratio of the concrete, where a relatively higher ratio facilitates the hydration of NS [68].

## 5. Influence of Nano Concrete Properties under High-Temperature Environment

**Figure 11:**
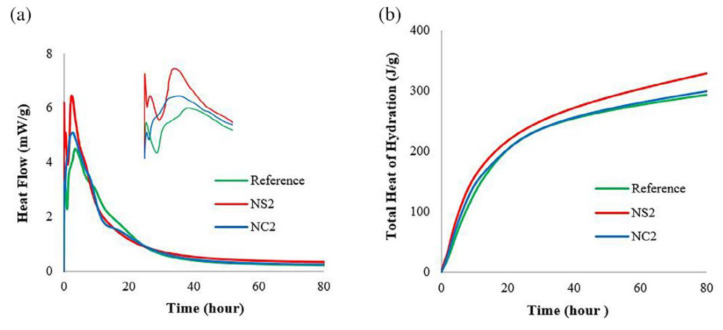
Hydration rate curve and cumulative heat of hydration of geopolymer slurry at 60 °C [69]. (**a**) Rate of hydration at 60 °C, (**b**) Total heat hydration at 60 °C.

**Figure 12:**
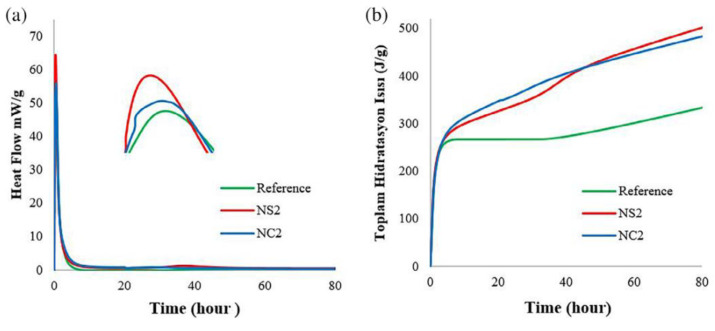
Hydration rate curve and cumulative heat of hydration of geopolymer slurry at 90 °C [69]. (**a**) Rate of hydration at 90 °C, (**b**) Total heat hydration at 90 °C.

Experiencing a fire or extreme high-temperature weather, through a series of reflections, the internal properties of concrete may change dramatically, by setting different high-temperature environments and simulating the state of concrete structure analysis, to derive the excellent medium difference of different nanomaterial admixture concrete, so as to develop the analysis [40,107]. Many experts and scholars have devoted themselves to the study of the fire resistance of nano concrete [108], which provides an important contribution to human production and life [109]. A comparison of the effects of geopolymers containing 2% NC and 2% NS exposed to temperatures of 60 °C and 90 °C follows. The cumulative heat-indicated reaction levels measured for 80 h at 60 °C and 90 °C show opposite results, as shown in Figure 11 and Figure 12. At a curing temperature of 90 °C, the addition of SiO_2_ nanoparticles resulted in a lower cumulative heat of reaction than the addition of NC until 40 h into the reaction, with the results showing a higher heat of reaction by the end of 80 h. The relative increase in the cumulative heat of reaction for NS addition can be seen in Figure 12, which shows peaks in the corresponding reaction rate curves between 35 and 45 h [69].

### 5.1. Residual Properties

Nanomaterials mixed with other media, such as fibres, will have different mechanical property degradations in high-temperature environments. L. Wu et al. [110] investigated the residual properties of carbon fibre-reinforced concrete after high temperatures with different NS-doping levels. The residual mechanical properties of NS carbon fibre-reinforced concrete after high temperatures were higher than those of ordinary concrete. After heating at 775 °C, the residual compressive strength, splitting strength and flexural strength of 0.25% carbon fibre and 1% NS concrete were 5.2%, 10.9% and 8.9% higher than those of ordinary concrete, respectively. The authors concluded that NS could effectively improve the mechanical properties of concrete after a high temperature and that the synergistic effect of NS and carbon fibre is the main factor for the improved mechanical properties of NS carbon fibre-reinforced concrete after a high temperature. A similar study was carried out by the authors Polat et al. [105], here using the singular material NS to improve the high-temperature resistance of the concrete, even when heated to 750 °C. The samples showed a 54% increase in compressive strength over the original concrete: a more striking finding than that of the previous author. The NS concrete specimens showed a faster loss of residual compressive strength of the concrete from 500 °C to 750 °C, with a 24% loss of compressive strength for the 500 °C samples: using a cement-mortar mixture, the temperature loss results were even more pronounced, as also reflected by the authors of Shah et al. [111]. In addition, for the different mechanical properties’ experiments, the loss of strength was also highly dependent on the size of the cement mixture, i.e., cubic (150 × 150 × 150), rectangular (10 × 10 × 40) and cylindrical (Ø150 × 300), etc.

Yonggui et al. [38] studied the compressive and splitting strengths of basalt fibre and NS in different temperature environments. By testing a large number of samples, it was concluded that the mechanical properties of concrete gradually decreased as the temperature continued to rise, and the authors concluded that the residual compressive strength of concrete between 25 °C and 600 °C was a quadratic function of temperature, with the compressive strength decreasing in the range of 400–600 °C to between 40–60%. The splitting tensile strength decreased significantly up to 400 °C, with decreases ranging from 20% to 60%, and smaller decreases above 400 °C. When the temperature was between 25 °C and 200 °C, the relative residual splitting strength increased linearly with temperature, confirming that the mechanical effect on nano fibre concrete is minimal in the 200 °C range. When the temperature exceeded 200 °C, the relative residual splitting strength was quadratic as a function of temperature. The splitting strength of concrete was less able to resist higher temperatures due to changes in the internal microstructure. Elsayd et al. [112] investigated the changes in mechanical and fire resistance properties using different nano-combinations for a room temperature environment of 25 °C and different high-temperature environments (200 °C, 400 °C, 500 °C, 600 °C, 700 °C and 800 °C). In the 200 °C range, both NS and nano-clay improved the mechanical properties of the concrete, gradually decreasing the residual compressive strength as the temperature increased. Exposure to 800 °C for 2 h resulted in a loss of up to 60% of the concrete’s strength. Compared to normal concrete, concrete configured with 3% NS and composite material (1% NS + 4% nano-clay) replacing cement, heated at 800 °C for 2 h after standard curing, increased the strength of the concrete by 19.8% and 14.7%, respectively, which can be used as an optimum percentage for the fire resistance properties of concrete. From a microscopic analysis, NS and NC-modified concrete specimens can still preserve better mechanical properties under different high-temperature environments, even when heated up to 800 °C. In response to the conclusions given by various scholars, the strength loss of nano concrete was not the same when heated to different high temperatures. No major differences were found between NS and NC in terms of the effect of mechanical properties in high-temperature environments. In addition to the mechanical compressive experimental tests and the splitting experimental tests, the review further expands on the flexural properties of nano concrete in a high-temperature environment.

NS and NC can improve the flexural properties of concrete in different temperature environments. By comparing 25 °C and 600 °C high temperatures, mixing NS improved the flexural strength and energy absorption capacity of the material. The flexural strength of NS mixed with 1.5% increased by 27% over the control sample in a room temperature environment, and by 21% after 600 °C high temperature. Unlike the NS modification, the addition of 3.0% NC was better at high temperatures than at room temperature. The flexural strength of concrete at an ambient temperature of 25 °C increased by 9% over the control sample and could be increased by 23% at 600 °C [113]. It is worth noting that the NS admixture at this point was only half of NC and the NS-modified concrete was able to provide better flexural performance results than NC. Cao et al. [114] compared the effect of NC and mixed micron calcium carbonate (MC) on the high-temperature properties of concrete. NC had the best high-temperature properties of the concrete samples, indicating that NC improved the high-temperature properties of the cement paste more significantly than MC, and NC particle size was beneficial to the development of high-temperature resistant mechanical properties. An overview of many articles found that many scholars are happy to start studies using NS to improve the high-temperature properties of concrete based on the activity and excellent modification ability of nano. From a durability point of view, the use of composites with different degrees of reaction, NC and NS, can result in a more neutral concrete with long-lasting performance excellence to meet the needs of the construction market.

### 5.2. Deformation Attenuation

**Figure 13:**
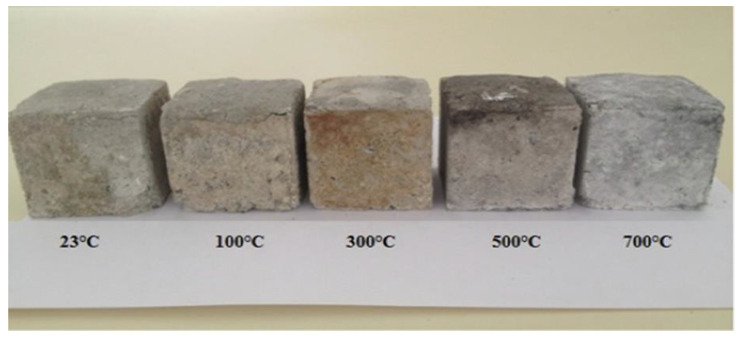
Variation in the appearance of concrete containing 2% NS after different temperatures [120].

**Figure 14:**
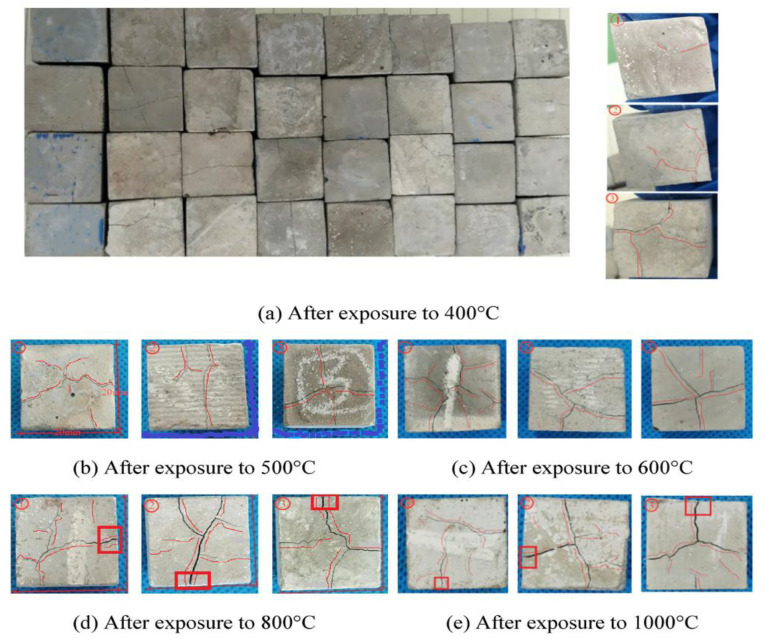
Exposed to different high-temperature environments [114]. ① Control samples, ② NS concrete samples, ③ MC concrete samples.

**Figure 15:**
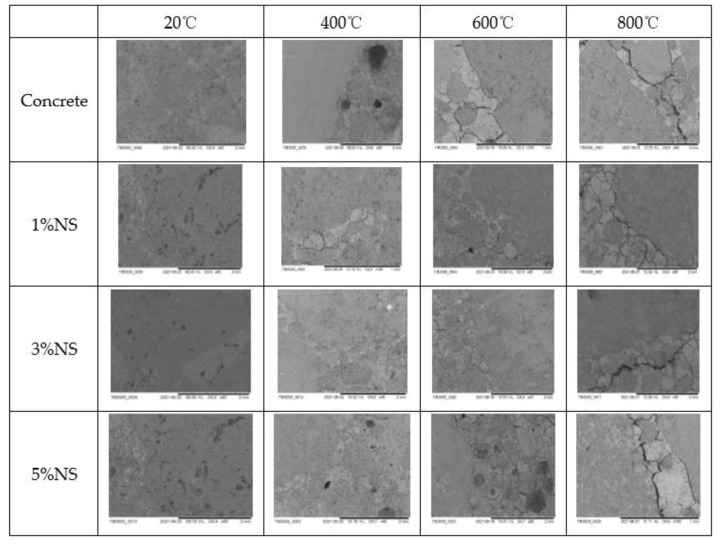
Appearance deformation of NS with different dosages after heating at 400 °C to 800 °C [121].

The ambient environment of NS and NC makes the interfacial transition zone of concrete denser and holds with the aggregates to form higher mechanical properties [115]. In contrast, in high-temperature environments, these voids gradually increase being amplified, creating micro-cracks until the interfacial state of the concrete is destroyed [31,116,117]. When concrete samples mixed with 3% NS and 15% alccofine were heated between 400 and 800 °C, the compressive strength of the concrete generally decreased, with the most significant loss of strength at 800 °C, and the surface colour changing from grey to white and finally to brown. The microscopic voids in the concrete became larger with increasing temperature [118]. Due to the fire and the increase in temperature, the residual compressive strength of NS and alccofine concrete at 200 °C lasting 4–8 h was higher than the strength of room-temperature concrete samples, and with no significant change in the surface of the concrete at 200 °C heated for 4 h and extended burning time, the surface of concrete became light grey. At 400 °C at 4 h of heating, the NS and alccofine concrete samples and control samples showed a light grey surface; at 8 h and 12 h of heating, the concrete surface turned dark grey; at 600 °C at high temperatures, the concrete surface also changed colour to brownish red. As the temperature increased to 800 °C, more significant numbers and widths of cracks appeared on the concrete surface. As the heating time continued to increase, the concrete colour changed from grey to white. From 600 °C to 1000 °C, aggregate decomposition was detected and the colour eventually turned red [119]. It increased linearly with increasing nano-doping and increasing temperature. Kantarci et al. [120] reported no significant change in the surface of concrete heated to 300 °C, which still contained a small amount of gloss, and gradually turned grey when heated from 500 °C to 700 °C, as shown in Figure 13. Cao et al. [114] investigated the effect of high-temperature environments from 200 °C to 1000 °C on the appearance of cracking of micron and NC-modified cement pastes. A visual analysis of the concrete surfaces showed that both NC and micron calcium carbonate (MC) affected their high-temperature properties. Within 600 °C, NC concrete samples and MC concrete samples produced less microcracking, while surface microcracking in the control concrete samples developed faster. At 800 °C high temperature, NC concrete samples produced more significant cosmetic microcracking than MC and the control concrete. The change in the appearance of cracks in the concrete exposed to different temperatures for 2 h is shown in Figure 14. Figure 15 shows SEM images after high-temperature heating: comparing the change in appearance for different NS dosages, the concrete shows a decrease in surface breakage after high-temperature heating. Temperatures reached 800 °C and the concrete was generally damaged, producing irregular cracks. NS had a positive effect on improving the high-temperature resistance of the concrete.

## 6. Discussion

Based on the effects of changing global climatic extremes and fire on concrete structures, NS and NC modified the concrete microscopically, with the microfilling of pores, volcanic ash and hydration resulting in a more dense internal structure. The mechanical properties of the modified concrete were analysed by a nano-analysis and the compressive properties, splitting properties and flexural properties were improved, respectively. The durability performance varied slightly with the tested parameters, and where environmental conditions were harsh, the NS and NC admixtures needed to be increased to achieve a good balance. Based on the existing basic research, the review article derives the residual properties and decay deformation of NC and NS-modified concrete after high-temperature environments. Based on the review, it was found that the mechanical properties of NS and NC-modified nano concrete after high-temperature effects were not significantly affected at 200 °C, the mechanical properties decreased at 400 °C and the strength loss of the concrete accelerated as it continued to increase. Some authors such as Khan et al. [122] evaluated the suitability of basalt fibres in both high-temperature and room-temperature environments, with the fibres of the composites retaining good mechanical properties under the influence of 850 °C and the basalt fibres preventing the spalling behaviour of the concrete. The effect of the mechanical properties of plain concrete of different grades at 925 °C ambient, as summarised by the authors Mathews et al. [123], decreased in the range of 39% to 46%. This shows that the added basalt fibres are an important way of stopping the behaviour change. Invoking the effects of nano-modification, the authors [110] used 0.25% carbon nano fibres in combination with 1% NS and obtained a very good high-temperature stability response. The NS and NC used alone required a certain amount of doping to be raised at the 200 °C to 800 °C studied, with the large number of scholars mixing NS alone at around 2% to 3% reflected in the review paper. This view is also reflected by the authors Polat et al. [105], where a single number of 2.5% nanomaterials improved the mechanical properties by 7% and 3% over normal concrete at 250 °C and 500 °C. Cao, Ming, et al. [124] studied the residual compressive properties of high-temperature calcium carbonate whisker concrete, with high temperatures causing CaCO3 and hydration products to decompose as a major factor in strength loss until 600 °C. The use of calcium carbonate whisker concrete from 600 to 700 °C had a complementary effect on the compressive and flexural properties of concrete [125]. NS acted to control C-S-H and hydrates more strongly, and compounding NS and NC into concrete will result in better high-temperature resistance and residual mechanical properties. After high-temperature heating the appearance, the experimental study of Kantarci et al. [120] found that the NS-modified appearance changed colour significantly more than that of Cao et al. [114]. In terms of high-temperature crack damage, NS concrete specimens changed more significantly than NC specimens, which was also confirmed by the authors Shah et al. [111]. From the nano-modification point of view, NS was more active than NC, which is a subjective modification. Analysed from a high-temperature performance point of view, NS concrete samples had more cracks in appearance above 400 °C than NC samples. NC had a relatively strong high-temperature stability. From an economic and application point of view, the use of composite NC and NS reduced the cost of engineering applications.

## 7. Conclusions

By reviewing this research for the properties of nanomaterial in concrete, the decay deformation pattern of NC and NS in a high-temperature environment was obtained and the following conclusions can be drawn:

- Both NC and NS cause a hydration reaction within the concrete and at the same time fill the voids in the concrete, making the internal structure denser, as can be seen from the SEM, TGA and Infrared Spectroscopy Analysis.
- The mechanical properties of the concrete incorporated with the composite nanomaterial NC+NS are significantly enhanced, with a corresponding increase in mechanical properties under different high-temperature environments. The results of the review study show that concrete samples heated at different high temperatures such as 200 °C, 400 °C, 600 °C and 800 °C have higher strengths than the control concrete. The mechanical strength of the concrete does not change significantly in the 200 °C range, and the strength decreases from 200 °C to 600 °C. After high-temperature heating at 800 °C, the mechanical strength of concrete loses about 60%.
- The dense NC and NS materials reduce the water permeability, depth of carbonation and self-shrinkage of the concrete, contributing to the development of durability.
- Mixing of NC and NS materials can improve the thermal insulation of concrete structures, with NS having a more pronounced effect than NC. This facilitates the development and application of special insulation features for concrete.
- After continuous high temperatures, from 200 °C to 600 °C, the surface colour of NC and NS concrete gradually changes from grey to white, and from 600 °C to 1000 °C the colour gradually darkens to brown. The surface of the concrete starts to show obvious cracks at 600 °C and the concrete is more severely damaged at 800 °C, with the phenomenon of peeling of the skin. Concrete mixed with NC and NS has more severe appearance damage than normal concrete due to being denser and more closed, with temperatures exceeding 600 °C. In addition, the structural damage to the concrete is more severe as the time at high temperatures increases.

## 8. Future Research

Mixed nanomaterials have been shown to be effective in improving the mechanical strength of concrete, and long-term mechanical property changes in concrete are worth tracking using NC and NS studies. The durability of nano concrete structures after high temperatures necessitates further research as concrete is exposed to high temperatures for long periods of time through global warming. New properties of nanostructures are being developed to withstand the sustained high temperatures of fires, shielding against radiation, and nanomaterials are expected to be used in critical structures. An overview drives the experimental research on composite nano (NC+NS) concrete and some new conclusions will be drawn.

## Figures and Tables

**Table 1:** Findings of mechanical properties of nano concrete.

Author	Year	Nanomaterials	Optimum Dosage (%)	Advance	Medium	Compressive Increase (%)	Splitting Increase (%)	Flexural Increase (%)	Influence Factor
[83]	2020	NC	2.0	Compressive, Flexural	NC	57.1	-	58.7	In total, 2.0% of NC particles have good cohesion to make the concrete denser, and 3.0% of NC agglomeration is severe and can lead to cracks.
[74]	2021	NC, NS	3.2	Compressive, Flexural	NS/NC + Steel fibres	10	-	21	The nucleation effect of nanomaterials and the development of an optimised internal organisation enhance the strength of concrete. The agglomeration of particles can occur in excessive amounts, weakening the interfacial transition zone.
[84]	2021	NC	1.5	Compressive, Splitting	NC	25	20	-	NC dosing above 1.5% will produce excessive hydration layers reducing strength.
[82]	2018	NC	0.5	Compressive, Splitting, Flexural	NC + Fly ash	57.2	36.9	45.2	NC reacts chemically with the elements in the cement paste, resulting in a significant increase in mechanical strength at 7 and 28 days. Greater modifying effect than the addition of fly ash.
[85]	2018	NS	2.5	Compressive, Splitting	NS	8.4	104.2	-	NS instead of cement mix concrete, smaller size fills the void of concrete to enhance the mechanical properties of the strength.
[81]	2020	NS	2.0	Compressive, Flexural	NS	9.7	-	17.1	Fine NS particles produce a hydration reaction that can be used for the restoration of old buildings.
[59]	2021	NS	2.0	Compressive, Splitting	NS + Silica fume	15.7	31.5	-	The volcanic ash effect of NS produces more C-S-H, making the interface transition zone dense and inhibiting the development of small cracks in concrete.
[86]	2019	NS	1.5	Compressive, Splitting	NS + Cellulose nano fibres	39	49	-	Transition zone between NS-modified cellulose nanofibres and gelling materials.
[87]	2018	NS	1.5	Compressive, Flexural	NS + Graphene oxide	43.2	-	42	NS particles are well dispersed and the hydration products resulting from the volcanic ash effect form a reticulated mix.
[58]	2019	NS	3.0	Compressive, Splitting, Flexural	NS + Polyethylene terephthalate	30.0	27.0	9.0	NS improves the interface transition zone between cement and PET aggregates.
[88]	2020	NS	1.5	Compressive, Splitting, Flexural	NS	-	-	14.82	Ultra-fine NS particles tighten the structure to produce more gel material.
[89]	2020	NS	3.0	Compressive	NS + Nano-CaO	23.4	-	-	NS modification has a denser microstructure and is accompanied by a self-healing ability.
[80]	2019	NS	3.0	Compressive	NS	38	-	-	The increase in strength is related to the water to glue ratio and the size of the NS particles, with the optimum range being between 2% and 5%. The hydrocolloid ratio increases as the voids become larger and the NS fills in the gaps.
[90]	2020	NS	2.0	Compressive, Flexural	NS	23.1	-	14.91	Promotes the hydration reaction of the cement, mixing 2.0% mass fraction of NS alone is the best result of the study.
[49]	2022	NS + NC	2.0	Compressive, Splitting, Flexural	NC, NS, NSC	8.8	4	9.3	NS optimises the void structure, NC makes the concrete structure denser and the effect of the two blends can synergistically improve dynamic and static mechanical properties.

**Table 2:** Findings of durability of nano concrete.

Author	Year	Nanomaterials	Optimum Dosage (%)	Advance	Influence Factor
[49]	2019	NC	3.0	Water absorption and hydrochloric acid resistance	NC reacts with the aluminate phase to produce more hydration products, reducing the water absorption of the concrete and increasing the hydrochloric acid resistance.
[82]	2018	NC	1.0	Water absorption and depth of penetration	NC increases the microscopic nucleation during hydration and thus reduces the void ratio of the concrete. Used together with fly ash, it results in a denser microstructure. Compared to the previous author, no hydrochloric acid resistance experiments are tested and the dosing of NC can be reduced to 1.0%.
[99]	2020	NC	1.0	Water absorption, chloride penetration and drying shrinkage	The addition of 1% NC interfacial bonding is better, mixed with a certain amount of slag and fly ash, and hydration increases to obtain excellent durability properties.
[100]	2020	NC	1.5	Impermeability	The high activity of nanoparticles, the increased surface effect and the hydration reaction make the concrete dense and improve the impermeability.
[96]	2019	NS	3.0	Depth of carbonation	The additional hydration products reduce the depth of carbonation of the concrete and resist penetration and attack by harmful substances.
[101]	2020	NS	3.0	Water absorption	The volcanic ash effect and microfilling effect of NS reduce the water absorption of concrete. NS is a good promoter of the modification of basalt fibre concrete.
[102]	2020	NS	3.0	Porosity and chloride ion permeation	NS with ultra-fine fly ash reduces chloride ion permeability from 53.83% to 71.45%. Both increase the density of the concrete due to the smaller particles. The authors set NS admixture levels from 0% to 4.5% and tests yield an optimum value of 3.0% for resistance to chloride ion permeation.
[103]	2020	NS	1.0	Water absorption and porosity	NS is used as a filler to fill the density of the concrete and to fill the voids in the internal matrix. A 1.0% replacement cement will achieve the required result.
[104]	2021	NS	0.75	Hydrochloric acid resistance	The gel produced by NS with the gelling material contributes to the development of durable properties. The Zn(OH)_2_ produced in the hydration reaction is able to resist the ingress of moisture.
[105]	2021	NS	2.5	Porosity and freeze-thaw resistance	The authors confirm through microscopic experiments that NS reflects with calcium hydroxide, producing a large number of C-S-H gel structures, densifying the microstructure and reducing the void fraction. Compared to the previous authors’ conclusions, the NS-doping is increased to 2.5% in order to obtain the best freeze-thaw resistance.
[68]	2020	NS + NC	1.0+3.0	Impermeability	The addition of NS and NC gives the carbon fibre concrete greater durability and a lower water–cement ratio of 0.4 for water penetration properties.
[106]	2022	NS, NC	2.0, 3.0	Water absorption and chloride ion penetration	In total, 2% NS reduces the water absorption of concrete to 58% and 2% NS can reduce the water absorption of concrete to 65–70%. The main reason for this action is the hydration of the nanoparticles filling the tiny voids.

## Data Availability

The data presented in this study are available upon request from the corresponding authors.
